# Evaluation of the Risk of African Swine Fever Virus Transmission at the Interface between Feral and Domestic Pigs in Lombardy, with a View to Establishing Preventive Measures for Domestic Pigs

**DOI:** 10.3390/pathogens12121462

**Published:** 2023-12-18

**Authors:** Stefania Calò, Marco Tironi, Veronica Cappa, Alessandra Scaburri, Stefano Francesco Perna, Mario Chiari, Massimo Marracci, Silvia Bellini

**Affiliations:** 1 Istituto Zooprofilattico Sperimentale della Lombardia e dell’Emilia-Romagna “Bruno Ubertini”, Via Bianchi, 9, 25124 Brescia, Italy; stefania.calo@izsler.it (S.C.); marco.tironi@izsler.it (M.T.); veronica.cappa@izsler.it (V.C.); alessandra.scaburri@izsler.it (A.S.); s.perna@izsler.it (S.F.P.); 2 Direzione Generale Welfare di Regione Lombardia, Unità Organizzativa Veterinaria, Piazza Città di Lombardia, 20124 Milan, Italy; mario_chiari@regione.lombardia.it; 3 Direzione Generale Agricoltura, Sovranità Alimentare e Foreste di Regione Lombardia, Unità Organizzativa Politiche Ittiche-Faunistico-Venatorie, Forestali e Montagna, Piazza Città di Lombardia, 20124 Milan, Italy; massimo_marracci@regione.lombardia.it

**Keywords:** African swine fever, control measures, interface domestic pigs, wild boar

## Abstract

African swine fever (ASF) continues to spread worldwide, and has reached multiple countries across Asia, the Caribbean, Europe, and the Pacific, representing a serious economic burden threatening pig health and welfare, as well as food security. The disease affects domestic pigs and wild boar, and in several European countries the disease is endemic in wild boars. The lack of vaccines or effective treatments highlights the importance of effective control measures used to keep domestic and feral pigs separated to prevent the spread of the virus. However, the study of the livestock–wildlife interface is quite complex and has many aspects to consider, including the uncertainty of wild-boar population data. In this study, we determined the risk of spread of the ASF virus at the interface between domestic pigs and wild boars using indicators that can indirectly indicate the presence of wild boars in order to target specific control measures in the highest risk areas. The results of the study were compared with those obtained by Pittiglio, in which the population data for wild boars was estimated using a geostatistical method and similar results were obtained. However, the present study used specific information relating to the wild-boar population and this allowed us to use fewer variables.

## 1. Introduction

A major global pandemic of African swine fever (ASF) is underway, and the strains that are currently circulating worldwide belong to genotype II. This virus strain is highly virulent and causes an acute fatal disease similar to a hemorrhagic fever in domestic pigs and wild boar. Despite the lack of zoonotic potential, the socio-economic impact is very high and trade restrictions associated with the occurrence of the disease can disrupt the regional and international trade in animals and animal products, with serious economic consequences for the swine sector [1]. Outbreaks caused by ASF virus (ASFV) genotype II are currently being reported in Africa, Europe, Asia, and the Pacific, as well as on the island of Hispaniola in the Americas. In the European Union (EU), this strain was introduced in 2014, and to date it has been reported in several EU member states, in most of which the disease is endemic in the wild-boar population and represents a constant threat to domestic pigs. 

Wild boars are one of the most intensively hunted species in Europe, and yet their numbers are increasing throughout Europe. However, estimating the size of a wild-boar population is challenging, and there is often a high degree of uncertainty as to the population’s size. Data on wild boar published by EFSA in 2018 show that the relative abundance is very high in many regions of Europe, and after almost 10 years of experience since the first occurrence of ASF in the EU, it seems clear that the current density of wild boar can facilitate the emergence and maintenance of the disease in the wild in several European countries [2]. 

In January 2022, ASFV genotype II was identified in wild boar for the first time in mainland Italy. The disease was detected in the northwest of the country, in a mountainous area on the border between the regions of Piedmont and Liguria. Since then, the disease has continued to spread in this area and, to date, about 900 cases have been reported in wild boar. After its introduction in Piedmont and Liguria, ASFV genotype II was also reported in Rome, and later in 2023 in mountainous areas of Campania and Calabria and in Lombardy. 

Northern Italy is an area of intensive pig farming, and Lombardy, a region bordering Piedmont, is home to about 50% of the national pig population. The Lombard pig sector is of economic importance for the entire country, also in terms of the processing industry, which aims to produce high-quality pork products. Therefore, the presence of an epidemic disease in pigs could adversely affect the entire pig sector. 

Prior to the introduction of ASF in mainland Italy, a study was carried out in Lombardy to identify the pig farms most at risk of ASF introduction and/or transmission. To this end, social network analysis (SNA) was used to analyze the trading patterns of pigs and to identify the farms that were central to the trading network in the region [3]. In fact, the movement of live animals and the means of transport, is the main risk for the spread of disease, especially in areas with high stocking-densities. In 2022, with the introduction of the infection in wild boar in a neighboring region, the risk scenario for Lombardy changed and the focus had to shift to the interface between domestic pigs and wildlife. In such a context, the pigs most exposed to the risk of ASF introduction are those kept on small and non-commercial farms, where pigs are kept for family consumption, and in outdoor pig-farming settings. In these husbandry systems, depending on the level of biosecurity, direct or indirect contact between wild and domestic pigs may occur. The literature reports that direct contact between domestic and wild pigs is uncommon [4,5]. However, a recent study in Serbia reported a frequency of 3.70 weekly visits of wild boars to pig farms, with a higher frequency observed in spring (7.5 visits). Wild boar visits to pig farms were mainly associated with the use of farm resources, such as food and water points [6]. Other studies have found that the proximity of forest to the farm and the distance between pig enclosures and houses (where farmers live) were factors that could influence wild-boar intrusions [7,8], as well as the presence of sows in estrus. In a study carried out in Romania, wild-boar density was identified as a risk factor for the occurrence of ASF in backyard farms. The proximity of crops to backyard farms, which are attractive to wild boar, or the provision of fresh feed to pigs have also been identified as significant risk factors for the occurrence of ASF in backyard farms in Romania. Indeed, given the resistance characteristics of ASFV, indirect contacts may also be effective in the transmission of the virus. The study of the livestock–wildlife interface and their interactions is quite complex and has many aspects to consider, including the uncertainty of wild-boar population data. 

The aim of this work was to determine the risk of ASF virus spread at the interface between domestic and feral pigs in Lombardy in order to identify the most exposed areas and where the risk of transmission could be reduced by applying appropriate control measures. For each municipality, the risk posed by the presence of wild boar in the area was assessed and then combined with the risk posed by the presence of domestic pigs. In Lombardy, data on wild boar were scarce, or at least their availability was not homogeneous throughout the region. Therefore, indicators that could indirectly indicate the presence of wild boar were used to determine the risk component linked to the presence of wild boar. Finally, the results of the study were compared with those obtained by Pittiglio [9] in 2018, in which the population density of wild boars was estimated by employing a geostatistical method based on a regression modelling using climatic and environmental covariates.

## 2. Materials and Methods

### 2.1. Description of the Area: Lombardy Region

Lombardy is a region in the north of Italy where intensive livestock farming and pig production is one of the most important livestock sectors. The region has an area of 23,863.1 km^2^, divided into 12 provinces and 1504 municipalities. According to the data recorded in the Regional Data Bank (RDB), the pig population is about 4 million in 6836 farms (2649 commercial, 4160 non-commercial and 27 free range), which represents 49.2% of the national pig population. The regional pig density is 177.2 animals/km^2^. The provinces where pig farming is most intensive are Brescia (2236 farms, 1,203,717 pigs), Mantua (707 farms, 1,071,732 pigs), and Cremona (501 farms, 942,419 pigs). The province of Brescia is the most populated, but the province of Cremona has the highest density of pigs, with 532.4 animals/km^2^. 

### 2.2. Calculation of Territorial Risk Related to the Presence of Wild Boar

The presence of wild boar in an area is a major risk factor for the transmission of certain pathogens between wild and domestic animals, and, in general, family or free-range farms are most at risk of their introduction. At present, it is difficult to find reliable census data on wild boar, so in order to determine the risk they may pose to domestic pigs, it is necessary to rely on the use of indicators that may indirectly indicate the presence of wild boar. Forested areas, nature parks, regional national parks, regional national reserves, special protection areas (SPAs), and special areas of conservation (SACs) provide favorable habitats for wild boar, so these areas are usually used as a proxy for the distribution of wild boar [10]. In this study, in addition to the presence of woodland, the following data were used to construct the risk indicator represented by the presence of wild boar:

- Results of trichinoscopic tests between 2017 and 2021 (source: Istituto Zooprofilattico Sperimentale data), taking into account that wild boar found dead or hunted must be tested for trichinosis. Georeferenced data is also available for these animals, which is useful for locating wild boar in municipal areas;
- Damage to agriculture caused by wild boar in 2019 and 2020 (source: Directorate-General for Agriculture, Food Sovereignty and Forestry);
- Road accidents caused by wild boar in 2020 and 2021 (source: Directorate-General for Agriculture, Food Sovereignty and Forestry).

The percentage of the municipal territory occupied by woodlands, nature parks, regional national parks, regional national reserves, SPAs and SACs was divided into three classes (0–50%, 50–80%, and 80–100%). At the municipal level, the combination of this information resulted in the wild-boar risk indicators shown in Table 1. For example, if the municipality has a high percentage of territory (≥80%) occupied mainly by woodlands, parks, reserves, SPAs and SACs, the risk of disease transmission was assessed as ‘medium’, but if the presence of wild boar was detected in the same territory, the risk of disease transmission was assessed as ‘high’. If the percentage of woodlands, parks, reserves, SPAs and SACs covers between 50% and 80% of the municipality, and the presence of wild boar was detected in at least two components, the risk was considered ‘high’.

### 2.3. Calculation of the Risk Associated with the Presence of Pigs

A total of 6913 pig establishments were selected from the RDB, including wild-boar farms and wild-boar hunting establishments (28). A total of 6836 pig farms were then analyzed, divided into the following farm types: non-commercial (family/personal consumption), commercial, and free range. Table 2 shows the number of farms and the number of animals by type.

**Figure 1:**
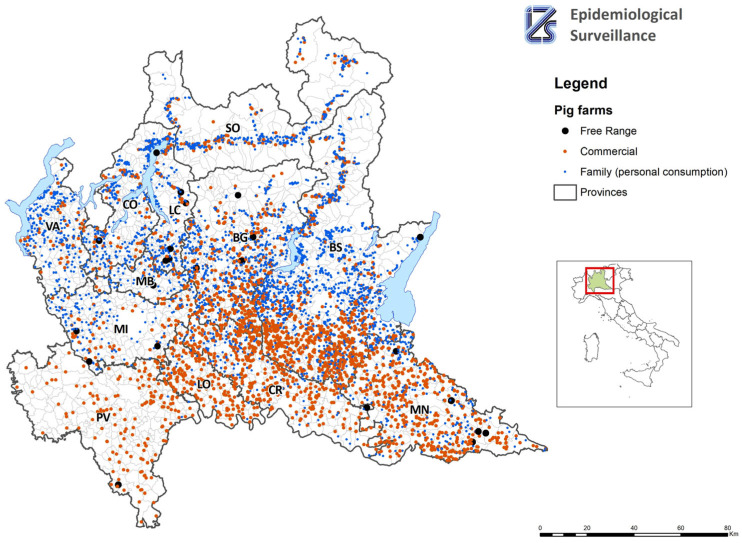
Map of pig farms in Lombardy.

Figure 1 shows the farm information on a map.

A check of the RDB data shows that 84.2% of the family farms have zero animals; this is a known problem for this production category, and it has an impact on the data analysis when constructing the corresponding indicator. Specifically, in the province of Pavia, family farms have zero animals, but this is because, as a precautionary measure, all animals on non-commercial farms have been culled, with a ban on restocking, due to the presence of ASF in Piedmont.

The risk of introducing ASF in pigs, determined per municipality, was classified as low, medium, or high. To calculate the indicator for each type of farm (personal consumption, commercial, and free range) and for each municipality,

1. The number of farms was calculated;
2. Densities were calculated according to the formula
  $${Farm\;density}_{municipality\;“i”}=\frac{Number\;of\;farms\;in\;municipality\;“i”}{Area\;of\;municipality\;“i”\;in\;{km}^{2}}$$
3. Farm densities by municipality and species were sorted in ascending order and then the tertiles of the distributions (33.3% and 66.6%) were calculated. For all farm types, tertiles were calculated using 95% of the distribution of the density indices of the municipalities;
4. Based on the tertiles and for each type of farm, the municipalities were classified as low, medium, or high risk, as shown in Table 3.

### 2.4. Overlapping of Pig–Wild-boar Risk for the Classification of Municipalities at Risk

The combined risk assessment obtained for the presence of wild-boar and pig farms was used to estimate the risk of disease transmission between wild and domestic pigs at the municipal level according to EFSA [10]. Four risk classes were created: low, medium-low, medium-high, and high (Table 4).

### 2.5. Software

The statistical analyses were performed in R version 4.2 [11]. The maps were created with ArcGIS 10.2 (ESRI, Redlands, CA, USA).

## 3. Results

### 3.1. Spatial Risk Linked to the Presence of Wild Boar

**Figure 2:**
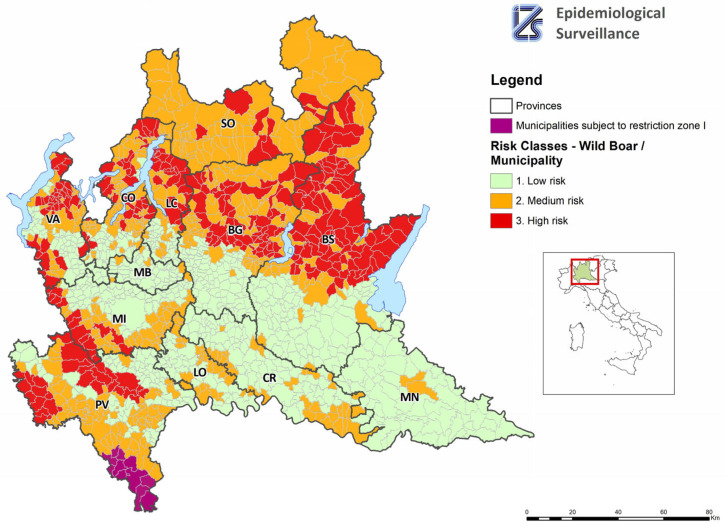
Map of municipalities at risk of African swine fever (ASF) introduction, exclusively due to the presence of wild boar.

Figure 2 shows the risk component related to the presence of wild boar. Municipalities in the province of Pavia that were already included in Restriction Zone I according to the European Commission’s Implementing Regulation 2022/1325 of 28 July 2022 are shown in purple on the map. These municipalities were already considered at risk of infection due to their proximity to the infected area of Piedmont and were therefore already subject to restrictive control measures. It is worth mentioning that the first two cases of ASF in wild boar were detected in this area in June 2023. In the same area, the infection was reported in domestic pigs in August 2023.

The indicator linked to the presence of wild boar, as established in this study, was compared with the methodology used by Pittiglio [9], using Kendall’s tau [12]. The test is statistically significant (tau = 0.34, *p*-value < 0.0001), showing a moderately positive concordance between the two classifications. It can be said that the two methods report the same level of risk of wild-boar presence at the municipal level. The wild-boar risk resulting from this work includes more specific information, in particular, hunted animals, damage to agriculture, and accidents caused by wild boar.

### 3.2. Territorial Risk Linked to the Presence of Pigs

As free-range farming is a high-risk area for the introduction of ASF, Table S1 shows the 27 free-range farms in Lombardy and their characteristics in relation to the percentage of the territory covered by woodland.

**Figure 3:**
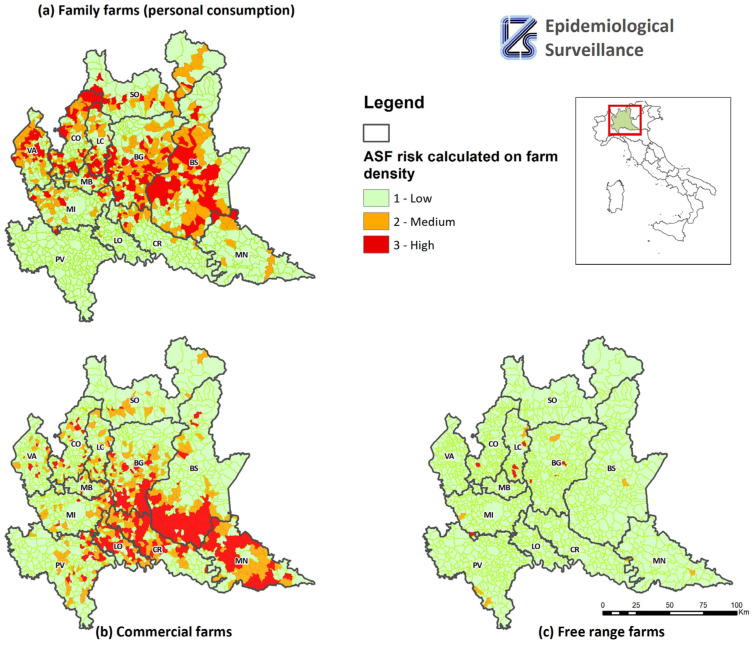
Map of municipalities at risk of ASF introduction based on farm density, by production type: (**a**) family farms (personal consumption), (**b**) commercial farms and (**c**) free-range farms.

Table 5 shows the number of municipalities by type of farm and by risk level related to the presence of pigs in the municipalities. In addition, Figure 3 shows the map of municipalities at risk by production type.

### 3.3. Overlapping of Domestic–Wild-Pig Risk for the Classification of Municipalities at Risk

**Figure 4:**
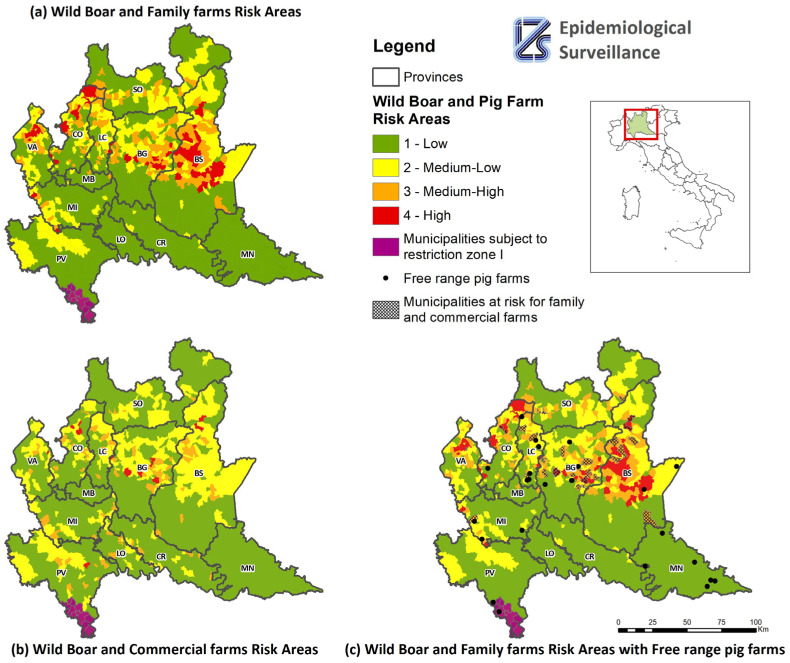
Map of municipalities at risk of ASF transmission between wild boar and domestic pigs, taking into account the overlap of the two risk-densities of farms and wild boar: (**a**) wild-boar and family farms risk areas, (**b**) wild-boar and commercial farms risk areas, (**c**) wild-boar and family farms risk areas with free-range pig farms.

Looking at the results of the indicator constructed using wild-boar and non-commercial farms (Figure 4a), there are 242 municipalities at medium-high or high risk (16.09%, 242/1504), of which 73 are at high risk (30.17%, 73/242), of which 34.25% are located in the province of Brescia (25/73). There are no high-risk municipalities in the provinces of Cremona, Lodi, Mantua, Monza, Pavia, and Sondrio. On the other hand, there are 169 municipalities at medium-high risk (69.83%, 169/242), of which approximately 25% fall within the provinces of Bergamo and Brescia (42/169 and 43/169). There are no municipalities at medium-high risk in the provinces of Cremona, Lodi, Mantua and Pavia.

In addition, the risk indicator was constructed considering commercial farms (Figure 4b). The results show that 105 municipalities are at medium-high or high risk (6.98%, 105/1504), of which 16 are at high risk (15.24%, 16/105), of which 56.25% are in the province of Bergamo (9/16); there are only two municipalities in the provinces of Pavia and Varese, respectively (6.3%, 1/16); there are none in the provinces of Cremona, Lecco, Lodi, Mantua, Milan, Monza, and Sondrio. On the other hand, there are eighty-nine municipalities at medium-high risk (84.76%, 89/105), of which 26.97% are in the province of Bergamo (24/89), and only four are in the province of Sondrio (4.49%, 4/89); there are no such municipalities in the provinces of Mantua and Monza.

To complete the analysis for the different types of pig farms, free-range farms were considered. There are 27 free-range pig farms in Lombardy, too few to be considered as a function of their density in the territory, and therefore they have been included on the map representing the wild-boar risk and family farms (Figure 4c). Six farms are located in medium-high-risk municipalities (22.22%, 6/27). Three of the municipalities at medium-high risk are located in the province of Lecco (50%, 3/6). Only one free-range farm is in restriction zone I (municipality of Ponte Nizza).

The creation of the commercial farm indicator was useful in identifying municipalities at risk for both commercial and family farms (Figure 4c). There are 43 such municipalities, represented on the map by a reticle, of which 48.84% (21/43) are in the province of Bergamo, 16.28% (7/43) in Brescia, and only one such municipality is in the province of Milan (2.33%, 1/43).

This information needs to be taken into account, as in these areas, the conditions could be in place for infection to spread from family farms to commercial farms. The two circuits cannot be considered completely separate, as they may have points of contact through company vehicles, slaughterhouses, or personnel working in contact with animals. The lists of municipalities with a medium-high or high risk of ASF virus transmission, as well as the list of the farms selected through SNA [3], have been sent to the veterinary authorities in order to intensify prevention and control measures on farms in the areas with the highest risk of ASF virus transmission.

## 4. Discussion

Currently there is no effective vaccine against ASF, and the only measures that can be adopted to manage this disease are based on prevention and early detection to avoid further spread of the infection. In 2022, with the introduction of the ASF in wild boar in Piedmont, the risk scenario for Lombardy changed and the focus had to switch to the interface between domestic pigs and wild boar in order to identify the most-exposed areas and apply appropriate disease control measures. 

In Lombardy, data on wild boars and their distribution are scarce, or in any case, their availability is not homogeneous throughout the regional territory; however, population data is necessary when undertaking control actions against a disease. To overcome this drawback, indicators have been constructed that attempt to best represent the presence of wild boar in the area, with the aim of identifying the municipalities in which the transmission of the virus from wild to domestic pigs is most likely, and to apply appropriate control measures. For each municipality, the risk posed by the presence of wild boar was assessed and then combined with the risk posed by the presence of domestic pigs. Based on the results, the municipalities were divided into four risk categories: low, medium-low, medium-high, and high (Table 4). The first case of ASF in wild boars, indeed, occurred on the border with Piedmont, in a municipality classified by the study as having a high risk of transmission, and the first ASF outbreak in Lombardy occurred in a small fattening farm in the restricted zone, in an area where the disease had already been identified in wild boars. However, it is worth remembering that, at the time of the study, pigs from family farms had already been slaughtered in that area in order to avoid the spread of the infection from feral to domestic pigs. In these farming conditions, after the elimination of pigs for household consumption, small fattening farms were, resultingly, the most exposed to the risk of introducing ASFV.

The indicator for the presence of wild boars established in this study was compared with the methodology applied by Pittiglio [9] using Kendall’s tau [12], and similar results were obtained with the two methods. However, the present study used specific information relating to the wild-boar population and this allowed us to use fewer variables while obtaining the same result. However, it should be considered that this information is generally not easy to collect on a large scale, but it can be useful when it is necessary to intervene with specific actions in smaller areas, in the absence of reliable data on the wild-boar population. The classification of the areas, together with the identification of high-turnover farms [3], can be useful to veterinary authorities in planning and prioritizing interventions such as raising the awareness and training of professionals; enhancing surveillance, together with the implementation and verification of biosecurity measures on farms; and, during hunting, preventively culling the herds most at risk.

Based on the results obtained, if we consider the indicator which includes the category of family farms, 83.93% of the municipalities in Lombardy are at low risk. This is not surprising, given that Lombardy is typically a region with intensive livestock farming. However, 34.25% of the municipalities at greatest risk for non-commercial farms are in the province of Brescia, which is the province where pig farming is most intensive. Therefore, municipalities with a high risk associated with both commercial and non-commercial farms were identified and considered to have an additional element of risk (Figure 4c). In these areas, we should pay particular attention to the farms identified by the SNA [3], where surveillance must be strengthened, and the correct application of biosecurity measures verified. Indeed, the two farming circuits are not completely independent; they have points of contact (e.g., trucks, slaughterhouses, and personnel), which implies that in these municipalities the infection has the potential to spread from wild boar to pigs on family farms and from these to commercial farms. In that event, given that these are densely populated areas, the infection could rapidly spread to the entire region and the rest of the country. In this regard, it is worth remembering that, in a previous study, we simulated the occurrence of ASF in wild boar in Lombardy [13]. The objective of the simulation was to evaluate the impact of the disease and the possible organizational and management consequences for veterinary services, given that infection in wild boars would still lead to the application of restrictive measures in domestic pigs. The infected zone was established in an area with a high density of pigs, in the province of Brescia, and approximately 150 km^2^ (12 km × 12 km) in size, which is plausible after the identification of an index case in wild boar. This zone contained 833 commercial farms and approximately 2000 non-commercial farms, for a total of 1,391,588 pigs and approximately 2,300,000 pigs marketed every year. This means that, even in the case of ASF in wild boars, given the restrictions also imposed on pig farms, there would be serious repercussions for the entire pig sector, both economically and in terms of animal welfare. The situation would certainly be worse in the event of spread to domestic pigs, where the costs due to the presence of the disease and the management of outbreaks would also have to be considered. One limitation of the study is the recording of the number of pigs on family farms in the regional database. A check showed 84.2% of the family farms as having zero animals, and this problem of missing data is well-known in this production category and has an impact on the data analysis when constructing the corresponding indicator. To overcome this problem, the indicator for this production category was constructed taking into account the number of farms. However, it should be borne in mind that some of these farms operate seasonally, and others genuinely have no animals, as some owners stop breeding pigs due to old age or other reasons, while they still retain the farm identification code.

## 5. Conclusions

The study of the livestock–wildlife interface and their interactions is quite complex and has many aspects to consider, including the uncertainty of wild-boar population data. Reliable population estimates are needed when measures are taken to manage animal populations or to control the spread of disease. The greater the difference is between the estimated population and the actual population size, the greater the error is in assessing the outcome of the strategy adopted. 

In this study, we determined the risk of the spread of the ASF virus at the interface between domestic and wild pigs, using indicators that can indirectly indicate the presence of wild boar. The results were compared with those obtained by Pittiglio in estimating wild-boar population density, and similar results were attained using a smaller number of variables. This study was conducted when Lombardy was still free from ASF; after its introduction, in June 2023, all cases (18) identified in wild boars occurred in municipalities classified as having a high risk of transmission; this seems to provide a field indication of the effectiveness of the developed methodology.

However, the ENETWILD consortium (www.ENETWILD.com, accessed on 20 March 2023) continues to work in this area to improve the quality of wild-boar data and to gain a better understanding of the interface between wild boar and domestic pigs in Europe, which is essential for analyzing the risks of spread of diseases shared between wildlife and livestock and evaluating the results of the implemented control strategies. 

## Figures and Tables

**Table 1:** Risk indicator (low, medium, or high) for ASFV transmission in the wild.

Percentage of Municipal Area Occupied by Woodlands and Parks, Reserves, SPAs, SACs	Trichinella (Hunted and Dead Wild Boars)	Accidents	Damage to Agriculture	Risk
>80%				Medium
>80%	X			High
>80%		X		High
>80%			X	High
>80%	X	X		High
>80%	X		X	High
>80%		X	X	High
>80%	X	X	X	High
50–80%				Medium
50–80%	X			Medium
50–80%		X		Medium
50–80%			X	Medium
50–80%	X	X		High
50–80%	X		X	High
50–80%		X	X	High
50–80%	X	X	X	High
<50%				Low
<50%	X			Low
<50%		X		Low
<50%			X	Low
<50%	X	X		Medium
<50%	X		X	Medium
<50%		X	X	Medium
<50%	X	X	X	Medium

For some municipalities there was no information on wild boar sufficient to classify the municipality according to risk. Where municipalities bordered or were located within medium- or high-risk municipalities, they were assigned the same risk level as the surrounding area.

**Table 2:** Number of pig farms and animals by type.

Type	Family/Non-Commercial	Commercial	Free Range
No. Farms	4160	2649	27
No. Average Animals (sd)	0.37 (0.98)	1593.9 (2670.6)	196.7 (948.4)
No. Animals			
0	3502 (84.2%)	571 (21.6%)	7 (26.9%)
1–4	651	181	4
5–30	7	258	13
>30	0	1645	3
>100	0	1550	1
>250	0	1481	1
>500	0	1371	1
>1000	0	1136	1

**Table 3:** Classification of municipalities into risk categories (high, medium, and low) according to the type of pig farms (personal consumption, commercial, and free range).

	Risk
Municipal density < 1st tertile	Low
1st tertile ≤ Municipal density ≤ 2nd tertile	Medium
Municipal density > 2nd tertile	High

**Table 4:** Classification of the overall risk of disease transmission between wild and domestic pigs at the municipal level.

Spatial Overlap		Wild Boar Risk		
		Low	Medium	High
Pig risk	Low	Low	Low	Medium-low
	Medium	Low	Medium-low	Medium-high
	High	Low	Medium-high	High

For the purposes of this study, municipalities with no pig farms and/or no wild boar were considered to be at low risk.

**Table 5:** Number of municipalities at risk, by type of farm.

	Type		
	Family	Commercial	Free Range
No. of farms	4160	2649	27
No. of municipalities with farms	939	770	26
Municipality risk			
Low	313	263	9
Medium	313	243	8
High	313	264	9

## Data Availability

Inquiries regarding data supporting can be directed to the corresponding author.

## Supplementary Materials

The following supporting information can be downloaded at: https://www.mdpi.com/article/10.3390/pathogens12121462/s1, Table S1: Free-range farms in Lombardy.
