# Supplementary material for: Evaluation of the Risk of African Swine Fever Virus Transmission at the Interface between Feral and Domestic Pigs in Lombardy, with a View to Establishing Preventive Measures for Domestic Pigs
Source: Pathogens. 2023 Dec 18;12(12):1462. doi: 10.3390/pathogens12121462 (PMC10748101; doi:10.3390/pathogens12121462)
Supplement: Supplementary file 1 [file pathogens-12-01462-s001.zip › TableS1.pdf]

FARM	SUB-CODE	TYPE	SPECIES	MUNICIPALITY	PROV	Agenzia di Tutela della Salute (ATS)	ANIMALS	% territory
A	4	Growing - finishing	Pigs	Montevecchia	LC	ATS OF BRIANZA	2	100%
B	7	Growing - finishing	Pigs	Besate	MI	ATS OF THE METROPOLITAN CITY OF MILAN	2	100%
C	2	Growing - finishing	Pigs	Tremosine Sul Garda	BS	ATS OF BRESCIA	0	100%
D	3	Breeding - closed loop	Pigs	Robecco Sul Naviglio	MI	ATS OF THE METROPOLITAN CITY OF MILAN	42	100%
E	3	Growing - finishing	Pigs	Introbio	LC	ATS OF BRIANZA	15	94%
F	4	Breeding - closed loop	Pigs	Roncobello	BG	ATS OF BERGAMO	4	93%
G	5	Breeding - closed loop	Pigs	Cremeno	LC	ATS OF BRIANZA	6	77%
H	3	Growing - finishing	Pigs	San Giuliano Milanese	MI	ATS OF THE METROPOLITAN CITY OF MILAN	6	75%
I	5	Growing - finishing	Pigs	Brianza Hill	LC	ATS OF BRIANZA	0	70%
J	9	Wild Boar Hunting Establishment	Wild boar	Sabbio Chiese	BS	ATS OF BRESCIA	0	66%
K	2	Growing - finishing	Pigs	Calvatone	CR	ATS OF THE PADANA VALLEY	0	63%
L	9	Breeding - open loop	Pigs	Valletta Brianza	LC	ATS OF BRIANZA	27	63%
M	1	Growing - finishing	Pigs	Ponte Nizza	PV	ATS OF PAVIA	0	50%
N	3	Breeding - open loop	Pigs	Cene	BG	ATS OF BERGAMO	5	48%
O	4	Breeding - closed loop	Pigs	Colico	LC	ATS OF BRIANZA	20	35%
P	5	Breeding - open loop	Pigs	Monza	MB	ATS OF BRIANZA	9	22%
Q	1	Wild Boar Hunting Establishment	Wild boar	Iseo	BS	ATS OF BRESCIA	3	26%
R	3	Breeding - closed loop	Wild boar	Torre De' Roveri	BG	ATS OF BERGAMO	43	18%
S	3	Breeding - closed loop	Wild boar	Torre De' Roveri	BG	ATS OF BERGAMO	13	18%
T	4	Breeding - open loop	Pigs	Villa Guardia	CO	ATS OF INSUBRIA	16	13%
U	1	Wild Boar Hunting Establishment	Wild boar	Rivanazzano Terme	PV	ATS OF PAVIA	5	13%
V	7	Growing - finishing	Pigs	Borgo Mantovano	MN	ATS OF THE PADANA VALLEY	0	12%
W	1	Growing - finishing	Pigs	Roncoferraro	MN	ATS OF THE PADANA VALLEY	27	10%
X	9	Breeding - open loop	Pigs	Quingentole	MN	ATS OF THE PADANA VALLEY	8	6%
Y	7	Breeding - closed loop	Pigs	Chignolo D'Isola	BG	ATS OF BERGAMO	18	5%
Z	1	Breeding - open loop	Pigs	Cavriana	MN	ATS OF THE PADANA VALLEY	4846	3%
AA	6	Growing - finishing	Pigs	Quistello	MN	ATS OF THE PADANA VALLEY	0	0%
